# How many steps/day are enough? For older adults and special populations

**DOI:** 10.1186/1479-5868-8-80

**Published:** 2011-07-28

**Authors:** Catrine Tudor-Locke, Cora L Craig, Yukitoshi Aoyagi, Rhonda C Bell, Karen A Croteau, Ilse De Bourdeaudhuij, Ben Ewald, Andrew W Gardner, Yoshiro Hatano, Lesley D Lutes, Sandra M Matsudo, Farah A Ramirez-Marrero, Laura Q Rogers, David A Rowe, Michael D Schmidt, Mark A Tully, Steven N Blair

**Affiliations:** 1 Walking Behaviour Laboratory, Pennington Biomedical Research Center, Baton Rouge, LA, USA; 2 Canadian Fitness and Lifestyle Research Institute, 201-185 Somerset Street West Ottawa, ON, K2P 0J2, Canada; 3 School of Public Health, Edward Ford Building (A27), University of Sydney, Sydney, NSW 2006, Australia; 4 Exercise Sciences Research Group, Tokyo Metropolitan Institute of Gerontology, 35-2 Sakaecho, Tokyo Itabashi, Tokyo 173, Japan; 5 Department of Agricultural, Food and Nutritional Science, 4-10 Agriculture/Forestry Centre University of Alberta, Edmonton, Alberta, T6G 2P5, Canada; 6 Department of Exercise, Health, and Sport Sciences, University of Southern Maine, 37 College Ave, Gorham, USA; 7 Department of Movement and Sport Sciences, Sint-Pietersnieuwstraat 25, Ghent University, B - 9000 Ghent, Belgium; 8 Centre for Clinical Epidemiology and Biostatistics, University of Newcastle, Callaghan, NSW 2308, Australia; 9 CMRI Diabetes and Metabolic Research Program, Harold Hamm Oklahoma Diabetes Center, University of Oklahoma Health Sciences Center, 1000 N. Lincoln Boulevard, Oklahoma City, OK, USA; 10 Tokyo Gakugei University, 4-1-1 Nukuikitamachi, Koganeisi, Tokyo 184-8501, Japan; 11 Department of Psychology, East Carolina University, Greenville, NC 27858, USA; 12 Centro de Estudos do Laboratório de Aptidão Física de São Caetano do Sul (CELAFISCS) & Agita São Paulo, São Caetano do Sul, Brazil; 13 Southern Illinois University School of Medicine, Department of Medicine, Springfield IL; 14 Department of Physical Education and Recreation, Rio Piedras Campus, University of Puerto Rico, San Juan, Puerto Rico; 15 Department of Medicine, Southern Illinois University School of Medicine, Springfield, IL, USA; 16 School of Psychological Sciences and Health, University of Strathclyde, Glasgow, Scotland, UK; 17 Department of Kinesiology, 115 Ramsey, University of Georgia, Athens GA 30602, USA; 18 Menzies Research Institute, Medical Science 1, 17 Liverpool Street, University of Tasmania, Hobart TAS 7000, Australia; 19 UKCRC Centre for Public Health (NI), Royal Victoria Hospital, Grosvenor Road, Queen's University, Belfast, Ireland; 20 Departments of Exercise Science and Epidemiology/Biostatistics, Arnold School of Public Health, University of South Carolina, Columbia, USA

## Abstract

Older adults and special populations (living with disability and/or chronic illness that may limit mobility and/or physical endurance) can benefit from practicing a more physically active lifestyle, typically by increasing ambulatory activity. Step counting devices (accelerometers and pedometers) offer an opportunity to monitor daily ambulatory activity; however, an appropriate translation of public health guidelines in terms of steps/day is unknown. Therefore this review was conducted to translate public health recommendations in terms of steps/day. Normative data indicates that 1) healthy older adults average 2,000-9,000 steps/day, and 2) special populations average 1,200-8,800 steps/day. Pedometer-based interventions in older adults and special populations elicit a weighted increase of approximately 775 steps/day (or an effect size of 0.26) and 2,215 steps/day (or an effect size of 0.67), respectively. There is no evidence to inform a moderate intensity cadence (i.e., steps/minute) in older adults at this time. However, using the adult cadence of 100 steps/minute to demark the lower end of an absolutely-defined moderate intensity (i.e., 3 METs), and multiplying this by 30 minutes produces a reasonable heuristic (i.e., guiding) value of 3,000 steps. However, this cadence may be unattainable in some frail/diseased populations. Regardless, to truly translate public health guidelines, these steps should be taken over and above activities performed in the course of daily living, be of at least moderate intensity accumulated in minimally 10 minute bouts, and add up to at least 150 minutes over the week. Considering a daily background of 5,000 steps/day (which may actually be too high for some older adults and/or special populations), a computed translation approximates 8,000 steps on days that include a target of achieving 30 minutes of moderate-to-vigorous physical activity (MVPA), and approximately 7,100 steps/day if averaged over a week. Measured directly and including these background activities, the evidence suggests that 30 minutes of daily MVPA accumulated in addition to habitual daily activities in healthy older adults is equivalent to taking approximately 7,000-10,000 steps/day. Those living with disability and/or chronic illness (that limits mobility and or/physical endurance) display lower levels of background daily activity, and this will affect whole-day estimates of recommended physical activity.

## Introduction

The profound and multiple benefits of living a physically active lifestyle extend to older adults and special populations (living with disability and/or chronic illness that may limit mobility and/or physical endurance) [1]. In reviewing their 2008 release of federal physical activity guidelines, the U.S. Advisory Committee Report concluded that, in addition to the well known cardiovascular and metabolic health benefits, there was "strong evidence" that physically active older adults have higher levels of functional health, lower risks of falling, and improved cognitive health [2]. A recent systematic review further confirmed that greater aerobic physical activity was associated with reduced risk of functional limitations and disability with age [3]. A systematic review of the benefits of physical activity for special populations is lacking, but it is presumed that similar returns are reasonable to expect.

Evidence-based guidelines for older adults communicate the benefits of a physically active lifestyle using frequency-, duration-, and intensity-based parameters. Similar to what is typically communicated to younger adults, public health physical activity guidelines promote at least 150 minutes/week of moderate-to-vigorous physical activity (MVPA) for older adults and include "brisk walking" as a primary example of an appropriate activity [3]. Variations on the message exist: the World Health Organization promotes at least 30 minutes of moderate intensity physical activity 5 days per week for older adults [4]. All older adults should avoid inactivity and some physical activity is considered better than none [5]; however, public health recommendations answer a pragmatic need to provide generalized guidance. Regardless of the message specifics, as framed, time- and intensity-based guidelines imply that this dose of physical activity should be taken *over and above *a baseline level which is yet to be quantified. This is problematic, since it is likely that this baseline level of non-exercise physical activity has been most susceptible to secular transitions in occupation in favour of desk jobs and reductions in physical demands of most other jobs, reliance on labour-saving devices to supplement or replace domestic tasks and other activities of daily living, dependence on motorized transportation, and an insidious and pervasive predilection for passive leisure time pursuits [6]. Since self-reported leisure time physical activity (specifically walking for exercise) increases in older adults with age [7], yet objectively monitored physical activity decreases [8], it is also likely that this baseline level of non-exercise physical activity is vulnerable to advancing age, disability, and chronic illness.

Step counting devices (i.e., pedometers and accelerometers) provide a means of objectively quantifying total daily activity, and their counting mechanisms are particularly sensitive to detecting the recommended intensities of walking believed to be associated with a host of healthful outcomes for older adults. Accelerometers can provide additional data with regards to time spent in various intensities of physical activity and inactivity in addition to providing step data. However, due to their relative expense and associated intensive data management requirements their use is typically limited to research. In contrast, simple and inexpensive pedometers, even if they are less sensitive to very slow walking [9], are more likely to be adopted for clinical and real world applications, including direct use by members of the public. Regardless of instrumentation choice, the utility of any step output is limited without the ability to translate public health guidelines in terms of steps/day.

## Methods

The Public Health Agency of Canada (PHAC) commissioned a literature review in February 2010 to inform an evidence-based approach to converting step count data into minutes of active time congruent with public health guidelines. An English-language search strategy identified 1,594 articles published since 2000 using the keywords (pedomet* or acceleromet*) and step* and ((physical activity) or walk*) within the following search engines: CINAHL, ERIC, MEDLINE, PsycINFO, SocINDEX, and SPORTDiscus. The list was subsequently reduced to 837 articles after duplicates, remaining non-English language articles, dissertations, non-peer reviewed articles, and those obviously not dealing with step-defined human physical activity were removed. Abstracts were reviewed, identified articles were assembled, and a report was written. Selected researchers from around the world with first-hand experience collecting step data in the relevant population were invited to critically review the report, identify any gaps or offer additional literature, check and verify data pulled from original sources, and intellectually contribute to this consensus article.

For the purposes of this article, we defined older adults as those older than 65 years of age, although much of the identified literature represents even older individuals. At times we considered studies that included at least some participants under 65 years of age, for example, as low as 50 years of age if the sample mean age was over 65 years of age. The definition of special populations was purposely quite broad and included studies of individuals living with disability and/or chronic illness that may limit mobility and/or physical endurance. Older adults with disabilities or chronic health problems, and frail older adults would more appropriately fit into the special populations category, however, this category is not necessarily defined solely by age. The final product herein is centred on the literature relevant to older adults and special populations with regards to: 1) normative data (i.e., expected values); 2) changes expected from interventions; 3) controlled studies that determine exact step-based conversions of timed behaviour; 4) computing a step translation of time- and intensity-based physical activity guidelines (e.g., steps/day associated with time in moderate-to-vigorous physical activity or MVPA); 5) directly measured steps/day indicative of minimal time in MVPA taken under free-living conditions; and, 6) steps/day associated with various health outcomes. Each section represents a 'mini-review.' At times the search strategy was exhaustive and the exact number of articles identified is presented under the appropriate heading below (e.g., direct studies of step-equivalents of physical activity guidelines). Where current reviews were identified (e.g., normative data), the findings were simply summarized herein and select original articles were referred to only to make specific points. Where appropriate, details of studies were tabulated. Any apparent inconsistencies in reporting within tables (e.g., instrument brand, model, manner in which participant age is reported, etc.) reflect reporting inconsistencies extracted directly from original articles. The child/adolescent [10] and adult populations [11] literature is reviewed separately.

## Results

### Normative data (expected values)

An early review of normative data from studies published between 1980 and 2000 [12] reported that we can expect 1) healthy older adults to take 6,000-8,500 steps/day (based on 10 studies identified that included adults age 50+ years with no specifically reported disabilities or chronic conditions); and 2) special populations to take 3,500- 5,500 steps/day (based on 8 studies identified representing a broad range of disabilities and chronic illnesses). The authors acknowledged that these expected values were derived from an amalgamation of few and disparate studies published at that time. Further, they anticipated that these normative data would and should be modified and refined as evidence and experience using pedometers to assess physical activity would inevitably continue to accumulate.

Since that time a number of studies focused on objectively monitored data have been published and the expected values for healthy older adults have been updated [13]. Specifically, 28 studies published between 2001 and 2009 focusing on adults ≥50 years of age not specifically recruited for illness or disability status were identified and assembled in a review article [13]. Step-defined physical activity ranged from 2,000- 9,000 steps/day, was (generally) lower for women than men, appeared to decrease over reported age groups, and was lower for those defined as overweight/obese compared to normal weight samples. A separate review article [14] summarized expected values from 60 studies of special populations including those living with heart and vascular diseases, chronic obstructive pulmonary disease or COPD, diabetes and dialysis, breast cancer, neuromuscular diseases, arthritis, joint replacement, fibromyalgia, and disability (impaired cognitive function/intellectual difficulties). Older adults with disabilities took the lowest number of steps/day (1,214 steps/day) followed by individuals living with COPD (2,237 steps/day). The highest number of steps/day (8,008 steps/day) were taken by individuals with Type 1 diabetes, followed by those living with mental retardation/intellectual disability (7,787 steps/day) and HIV (7,545 steps/day). It is apparent that special populations, broadly defined, include those whose disability and/or chronic illness may or may not limit their mobility and/or physical endurance.

Tudor-Locke and Bassett [15] originally proposed a graduated step index to describe pedometer-determined habitual physical activity in adults: 1) < 5,000 steps/day (sedentary); 2) 5,000-7,499 steps/day (low active); 3) 7,500-9,999 steps/day (somewhat active); 4) ≥ 10,000-12,499 steps/day (active); and 5) ≥12,500 steps/day (highly active). These incremental categories were reinforced in a second review in 2008 [16]. Recognizing a considerable floor effect (i.e., insensitivity to the range of activity levels below the lowest threshold) when applied to low active populations, Tudor-Locke et al. [17] suggested that the original sedentary level could be further divided into two additional incremental levels: < 2,500 steps/day (basal activity) and 2,500- 4,999 steps/day (limited activity). As it stands, this graduated step index represents an absolute classification scheme. For example, it does not take into consideration that advancing age or the presence of chronic disease/disability generally reduces levels of activity. As such older adults and special populations will be always compared to younger populations with less disability or illness.

Table 1 displays those studies of free-living behaviour reporting the percent meeting select step-defined cut points in older adults and special populations (specifically individuals living with HIV [18], as no other relevant article was located on special populations). These limited studies indicate that achieving > 10,000 steps/day is likely to be challenging for some (e.g., those taking less than 2,500 steps/day), but not necessarily impossible for all older adults (e.g., those taking more than 9,000 steps/day).

**Table 1:** Studies of free-living behaviour reporting percent meeting select step-defined cut points in older adults

First Author	Sample Characteristics	Instrument	Monitoring Frame	Cut points used	% Meeting Specified Cut Point
Tudor-Locke [37] 2002 Canada	6 men, 12 women; Community dwelling older exercisers; 59-80 years	Yamax Digiwalker SW-200, Yamax Corporation, Tokyo, Japan	9 days	10,000	50% never achieved 10,000 steps on any day of the monitoring frame

Newton [58] 2006 UK	54 women; primary biliary cirrhosis patients 63.0 ± 9.4 years	Actigraph MTI Health Services, USA	6 days	Adult Graded Step Index	24% > 10,000

Rowe [55] 2007 UK	29 men, 60 women community dwelling 60+ years	Yamax Actigraph	7 days	10,000	9.6% of days > 10,000

Ewald [88] 2009 Australia	322 men, 362 women; community-dwelling, urban; 55 to 85 years	Yamax Digwalker SW-200	7 days	8,000 [84]	Overall: 42% > 8,000 55-59 year olds: 62% 80+ year olds: 12%

In summary, the updated normative data indicate that 1) apparently healthy older adults average 2,000-9,000 steps/day, and 2) special populations average 1,200-8,800 steps/day. The very broad ranges of habitual activity reflect the natural diversity of abilities common to older adults and special populations, especially given that not all chronic conditions are expected to significantly impact physical mobility and/or endurance. Further, individuals with a chronic illness are not necessarily "older," further exacerbating this wide variability. Normative data continue to be published. These normative data provide an important set of reference values by which individual or group data can be compared to assumed peers. Use of a graduated step index permits classification of older adults and special populations by multiple step-defined physical activity categories. On-going surveillance of step-defined physical activity is required to track progress, identify areas of concern, and evaluate the efficacy and effectiveness of public health strategies. The next step will be to improve understanding about determinants of step-defined physical activity, including the impact of disability and chronic illness on contexts (e.g., occupation, retirement, transport, leisure, home, living arrangements, etc.) where older adults and special populations accumulate (or do not accumulate) steps, especially those of at least moderate intensity (defined below).

### Interventions

Although three previous reviews have documented the effects of pedometer-based programming on physical activity [19-21], weight loss [19,20], and blood pressure [19] in samples that have included older adults and special populations, no review has specifically examined intervention effects in either of these groups at this time. Yet these are the groups that may be most attracted to pedometer-based programming. Participants in pedometer-based community interventions delivered in Ghent, Belgium [22] and Rockhampton, Australia [23] were more likely to be older than younger. Although no actual pedometer data were reported, a library-based pedometer loan program delivered in Ontario, Canada reported that older adults (55+ years of age) were more likely to participate than other age groups.

Table 2 presents details from 13 identified pedometer-based physical activity intervention studies that have focused on older adult samples ranging in age from 55 to 95 years. The majority of participants were community-dwelling, however a few studies reported interventions with older adults living in continuing care [24], congregate housing [25], or assisted living situations [26]. Interventions have lasted from 2 weeks [24] to 11 months [27] in duration. The mean baseline step-defined physical activity was 4,196 steps/day (weighted mean = 3,556 steps/day); a value that is considered representative of sedentary populations [15]. The mean delta (i.e., difference between pre- and post-intervention) was 808 steps/day; adjusted for sample size the weighted mean delta was 775 steps/day. In comparison, a change of 2,000-2,500 steps/day is typical of pedometer-based interventions in younger adults [19,21]. Study-specific effect sizes (Cohen's D) were computed where necessary data were provided in the original article, and these also appear in Table 2. Overall, the weighted effect size was 0.26 (generally considered a small effect). This effect size is also smaller than what is expected in younger adult populations (i.e., 0.68) [21].

**Table 2:** Pedometer -based physical activity intervention studies with older adults

Reference	Sample	Intervention duration; study duration and design	Instrument	Intervention Group Baseline Steps/day	Intervention Group Immediately Post-Intervention Steps/day	Delta Steps/day	Cohen's D
Conn [89] 2003 USA	65-96 years; community-dwelling; 190 participants	3-month intervention; 3-month randomized controlled trial	Yamax Digi-Walker	2,773 ± 1,780	2,253 ± 1,394	-520	-0.33

Croteau [26] 2004 USA	68-95 years; living in assisted living; 15 participants	4-week intervention; 4-week quasi-experimental	Yamax Digi-Walker SW-200	3,031 ± 2,754	2,419 ± 2,296	-612	-0.24

Jensen [90] 2004 USA	60-75 years; community -dwelling; 18 participants	3-month intervention; 3-month quasi-experimental	Accusplit, San Jose, CA	4,027 ± 2,515	5,883 ± 3,214	1,856	0.65

Croteau [25] 2005 USA	60-90 year olds; living in congregate housing or community-dwelling; 76 participants	4- month intervention; 4-month quasi-experimental	Accusplit AX120, San Jose, California	4,041 ± 2,824	5,559 ± 3,866	1,518	0.45

Croteau [91] 2007 USA	55-94 years; community-dwelling; 147 participants	12-week intervention; 12-week quasi-experimental	Yamax Digi-Walker SW-200 (Yamax Corporation, Tokyo, Japan)	4,963	≅ 6,200	≅ 1,237	N/A

Sarkisian [92] 2007 USA	≥ 65 years; community-dwelling; 46 participants	7-week intervention; 7-week quasi-experimental	Digiwalker (Yamax DW-500, New Lifestyles, Inc., Kansas City, MO)	3,536 ± 2,280*	4,387 ± 2,770*	851	0.34

Wellman [93] 2007 USA	Mean 74.6 years; community-dwelling; 320 participants	12-week intervention; 12-week quasi-experimental	NR	3,110 ± 2,448	4,183 ± 3,257	1,073	0.38

Rosenberg [24] 2008 USA	74-92 years; living in continuing care retirement community; 12 participants	2 week intervention; 3-week quasi- experimental	Accusplit AH120M9, Pleasanton, CA	3,020 ± 1,858	4,246 ± 2,331	1,226	0.59

Culos-Reed [94] 2008 Canada	46-83 years; community-dwelling; 39 participants	8-week intervention; 8-week quasi-experimental	NR	5,055 ± 1,374	5,969 ± 1,543	914	0.63

Fitzpatrick [95] 2008 USA	Mean 75 years; attending senior centers; 418 participants	4-month intervention; 4-month quasi-experimental	Accusplit, San Jose, CA	2,895 ± 2,170	3,743 ± 2,311	848	0.38

Opdenacker [27] 2008 Belgium	≥ 60 years; community-dwelling; 46 intervention participants	11-month intervention; 23-month randomized controlled trial	Yamax Digiwalker SW-200, Yamax Corporation, Tokyo, Japan	7,390 ± 2,693**	7,465 ± 3,344**	75	0.02

Sugden [96] 2008 U.K.	70-86 years; community-dwelling; 54 participants	12-week intervention; 12-week randomized controlled trial	Omron HJ-005	2,895	NR	N/A	N/A

Koizumi [97] 2009 Japan	60-78 years; community-dwelling; 34 intervention participants	12-week intervention; 12-week randomized controlled trial	Kenz Lifecorder, Suzuken Company, Nagoya, Japan	7,811 ± 3,268	9,046 ± 2,620	1,235	0.42

Steps/day presented as mean ± SD unless otherwise noted; *reported as steps/week in original article; divided by 7 days here; **SD calculated from reported SE

Table 3 displays details from identified pedometer-based physical activity intervention studies in special populations that have reported any steps/day data. Specifically, we located 10 studies in cancer populations, three in COPD populations, two in coronary heart disease and related disorders, 15 in diabetes populations, and 3 in populations with joint or muscle disorders. Across conditions, intervention durations have ranged from 4 weeks [28,29] to 12 months [30,31]. Some researchers have chosen to intervene using a pedometer but to assess outcomes using an accelerometer [31-36]. Delta values and effect sizes were computed for each study where requisite data were reported. Additionally, we have presented unweighted and weighted (taking into consideration sample size) deltas and effect sizes by condition. Mean weighted deltas ranged from 562 steps/day for COPD to 2,840 steps/day for coronary heart disease and related disorders. Weighted effect sizes ranged from 0.06 (small) for COPD to 1.21 (large) for coronary heart disease and related disorders. Across conditions, unweighted mean delta and effect size were 2,072 steps/day and 0.64, respectively. Weighted values were 2,215 and 0.67 (medium), respectively.

**Table 3:** Pedometer - based physical activity intervention studies with special populations

Reference	Sample	Intervention duration; study duration and design	Instrument	Intervention Group Baseline Steps/day	Intervention Group Immediately Post-Intervention Steps/day	Delta Steps/day	Cohen's D
***Cancer***							

Wilson [98] 2005 USA	Adult breast cancer survivors; 22 intervention participants	8-week intervention; 8-week quasi-experimental	NR	4,791	8,297	3,506	N/A

Pinto [32,33] 2005, 2009 USA	Adult breast cancer survivors; 43 intervention participants	12-week intervention; 9-month randomized controlled trial	Intervention: pedometer (Yamax Digiwalker) Assessment: accelerometer (Caltrac, Muscle Dynamics, Torrance, CA)	4,471.7 ± 5,196.1	14,571.5 ± 9,489.5	10,100	1.38

Vallance [99] 2007 Canada	Adult breast cancer survivors; 94 print materials, 94 pedometer only, 93 pedometer with print materials, 96 standard recommendation	3-month intervention; 6-month randomized controlled trial	Digi-Walker SW-200 PED (New Lifestyles Inc., Lee's Summit, MO)	8,476 ± 3,248 (Pedometer only) 7,993 ± 3,559 (Pedometer with print materials)	8,420 ± 5,226 (Pedometer only) 7,783 ± 3,048 (Pedometer with print materials)	-210	-0.06

Irwin [100] 2008 USA	Adults with early stage breast cancer; 37 intervention participants	6-month intervention; 6-month randomized controlled trial	NR	5,083 ± 2,313 (based on n = 37)	6,738 ± 2,958 (based on n = 34)	1,655	0.63

Pinto [34] 2008 USA	Breast cancer survivors; 25 intervention participants	12-week intervention; 24-week quasi-experimental	Intervention: pedometer (Yamax Digiwalker) Assessment: accelerometer (Biotrainer-Pro, Individual Monitoring Systems, Baltimore, MD)	No pre-intervention steps data reported but week one mean steps/day = 515.8 ± 470.8	1,695.4 ± 1,221.3	1,180	1.39

Matthews [35] 2007 USA	Breast cancer survivors; 13 intervention participants	12-week intervention; 12-week randomized comparative trial	Intervention: pedometer (Brand NR) Assessment: Manufacturing Technology Actigraph (MTI, Fort Walton Beach, FL, USA)	7,409.4 ± 2,791.1	8,561.8 ± 2887.3	1,152	0.41

Blaauwbroek [101] 2009 The Netherlands	Adult survivors of childhood cancer; 38 intervention participants	10-week intervention; 36-week quasi-experimental	Yamax digiwalker SW-200	7,653 ± 3,272	11,803 ± 3,483	4,150	1.23

Mustian [28] 2009 USA	Mixed cancer type patients receiving radiation; 19 intervention participants	4-week intervention; 3-month randomized controlled trial	NR	7,222 ± 2,691	11,200 ± 5,851	3,978	0.93

Swenson [30] 2010 USA	Breast cancer patients receiving chemotherapy; 36 intervention participants (subsample of larger randomized trial)	12- month intervention; 12-month quasi-experimental study conducted within a larger randomized trial	Walk 4 Life LS2500 (Walk 4 Life, Inc.)	No pre-intervention steps data reported but week one mean steps/day = 7,453 ± 2,519	9,429 ± 3,488	1,976	0.66

					Unweighted mean	2,743	0.73
					
					Weighted mean	2,139	0.51

***Chronic obstructive pulmonary disease (COPD)***							

De Blok [102] 2006 The Netherlands	Adults with COPD; 8 intervention participants	9-week intervention; 9 week randomized controlled trial	Yamax Digi-Walker SW-200 (Tokyo, Japan)	2,140	3,927	1,787	N/A

Hopses [103] 2009 The Netherlands	Adults with COPD; 18 intervention participants	12-week intervention; 12-week randomized controlled trial	Digiwalker SW-2000 (Yamax, Tokyo, Japan)	7,087 ± 4,058	7,872 ± 3,962	785	0.20

Nguyen [36] 2009 USA	Adults with COPD; 8 self-monitored (SM), 9 coached (C)	6-month intervention; 6-month randomized comparative trial of cell-phone supported pedometer programs	Intervention: Omron HJ-112 (Omron Healthcare, Bannockburn, IL, USA) Assessment: Stepwatch 3 Activity Monitor (SAM; OrthoCare Innovations, Washington, DC, USA)	SM: 5,229 ± 3,021* C: 6,692 ± 3,021*	SM: 5,838 ± 3,100* C: 5,675 ± 3,021*	SM: 609 C: -1,017	SM: 0.02 C: -0.34

					Unweighted mean	541	0.02
					
					Weighted mean	562	0.06

***Coronary heart disease and related disorders***							

VanWormer [104] 2004 USA	Adults with coronary artery disease; 22 intervention participants	17-week intervention; 17-week quasi-experimental	NR	6,520.10 ± 2,926.99	8,210.24 ± 2,534.91	1,690	0.62

Izawa [105] 2005 Japan	Adult myocardial infarction patients completing 6 months of cardiac rehabilitation; 24 intervention participants	6-month intervention; 12-month randomized controlled trial	Kenz Lifecorder, (Suzuken, Nagoya, Japan)	6,564.9 ± 1,114.6	10,458.7 ± 3,310.1	3,894	1.76

					Unweighted mean	2,792	1.29
					
					Weighted mean	2,840	1.21

***Diabetes and related disorders***							

Tudor-Locke [29] 2001 Canada	Adults with type 2 diabetes; 9 intervention participants	4-week intervention; 4-week quasi-experimental	Yamax Digiwalker SW-200	6,342 ± 2,244	10,115 ± 3,407	3,773	1.34

Tudor-Locke [106] 2004 Canada	Adults with type 2 diabetes; 24 intervention participants	16-week intervention; 24-week randomized controlled trial	Yamax SW-200, (Yamax Corporation, Tokyo, Japan)	5,754 ± 2,457	9,123 ± 4,539	3,369	0.96

Araiza [107] 2006 USA	Adults with type 2 diabetes; 15 intervention participants	6-week intervention; 6-week; randomized controlled trial	Yamax Digiwalker SW-701 (New Lifestyles, Kansas City, MI)	7,220 ± 2792	10,410 ± 4,162	3,190	0.92

Engel [108] 2006 Australia	Adults with type 2 diabetes; 30 coaching intervention, 24 pedometer intervention	6-month intervention; 6-month randomized comparative trial	Yamax Digi-Walker-700	NR	averaged 7,296 ± 2,066 during intervention	N/A	N/A

Richardson [109] 2007 USA	Adults with type 2 diabetes; 17 lifestyle goals, 13 structured goals	6-week intervention; 6-week comparative trial of two types of pedometer goal-setting strategies	Omron HJ-720IT (beta test version)	Lifestyles goals: 4,157 ± 1,737 Structured goals: 6,279 ± 3,306	Lifestyles goals: 5,171 ± 1,769 Structured goals: 6,868 ± 3,751	Lifestyles goals: 1,014 Structured goals: 589	Lifestyles goals: 0.58 Structured goals: 0.17

Bjorgaas [110] 2008 Norway	Adults with type 2 diabetes; 19 intervention participants	6-month intervention; 6-month randomized controlled trial	Yamax Dig-Walker ML AW-320, Yamax Corp, Tokyo, Japan	7,628 ± 3,715	8,022 ± 3,368	394	0.11

LeMaster [31] 2008 USA	Adults with diabetic peripheral neuropathy; 41 intervention participants	12-month intervention; 12-month randomized controlled trial	Intervention: Accusplit Eagle 170 (Pleasanton, CA) Assessment: Stepwatch 3 (Orthocare Innovations, Washington, DC)	3,335 ± 1,575*	3,183 ± 1,537*	-152	-0.10

Cheong [111] 2009 Canada	Adults with type 2 diabetes; 19 pedometer-only intervention (P); 19 pedometer and low glycemic index food intake intervention (PGI)	16-week intervention; 16-week randomized comparative trial	NR	P: 5,721 ± 2,232* PGI: 5,251 ± 1,944*	P: 8,527 ± 3,374* PGI: 9,381 ± 5,187*	P: 2,806 PGI: 4,130	P: 1.00 PGI: 1.16

Johnson [112] 2009 Canada	Adults with type 2 diabetes; 21 Enhanced program, 17 Basic program	12-week randomized comparative evaluation of two types of pedometer programs	Digi-Walker SW-200, (Yamax, Kyoto, Japan)	All participants: 8,948 ± 3,288	All participants: 10,485 ± 4,264**	1,685	0.44

Kirk [113] 2009 U.K.	Adults with type 2 diabetes; 42 in-person intervention (IP), 40 written form intervention (WF)	6-month intervention; 12-month randomized controlled trial	ActiGraph GT1M (ActiGraph LLC, Pensacola, FL, USA)	IP: 6,600 ± 2,700 WF: 5,500 ± 2,300	IP: 6,500 ± 2,300 WF: 5,300 ± 2,300	IP: -100 WF: -200	IP: -0.04 WF: -0.09

Newton [114] 2009 New Zealand	Adolescents with type 1 diabetes; 34 intervention participants	12-week intervention; 12-week randomized controlled trial	NR	Median 11,242	Median 10,159	N/A	N/A

Tudor-Locke [115] 2009 Canada	Adults with type 2 diabetes; 157 professional-led (PRO), 63 peer-led (PEER) participants	16-week intervention; 16-week quasi-experimental comparison of program delivery	Yamax SW-200, (Yamax Corporation, Tokyo, Japan)	PRO: 3,980 ± 2,189 PEER: 4,396 ± 2,045	PRO: 7,976 ± 4,118 PEER: 8,612 ± 3,202	PRO: 3,996 PEER: 4,216	PRO: 1.27 PEER: 1.61

Vincent [116] 2009 USA	Adults with type 2 diabetes; 9 intervention participants	8-week intervention; 8-week randomized controlled trial	NR	4,175	7,238	3,063	N/A

De Greef [72] 2010 Belgium	Adults with type 2 diabetes; 20 intervention participants	12-week intervention, 12-week randomized controlled trial	Yamax DigiWalker SW200	7,099 ± 4,208	8,024 ± 5,331	925	0.19

Diedrich [117] 2010 USA	Adults with type 2 diabetes; 11 intervention participants	3-month intervention; 3-month quasi-experiment	Yamax Digiwalker SW-200	4,145 ± 2,929***	6,486 ± 2,766***	2,341	0.82

					Unweighted mean	2,061	0.65
					
					Weighted mean	2,405	0.78

***Joint or muscle disorders***							

Talbot [118] 2003 USA	Adults with knee osteoarthritis; 17 walking plus education program	12-week intervention; 12-week randomized comparative trial of a self-management education program with and without walking program	New Lifestyles Digi-walker SW-200 (Yamax, Tokyo, Japan)	3,519 ± 2,603	4,337 ± 2,903	818	0.30

Kilmer [119] 2005 USA	Adults with neuromuscular disease; 20 intervention participants	6-month intervention; 6-month quasi-experimental home-based activity and dietary intervention	NR	≅ 4,600 (from figure)	≅ 5,900 (from figure)	N/A	N/A

Fontaine [120] 2007 USA	Adults with fibromyalgia syndrome; 14 intervention particpants	12-week intervention; 12-week randomized comparative trial	Accusplit Eagle Activity Pedometer (San Jose, CA)	2,337 ± 1,598*	3,970 ± 2,238*	1,633	0.85

					Unweighted mean	1,226	0.57
					
					Weighted mean	1,186	0.55

Note: Values are means ± SD unless otherwise stated, personal communication with Fontaine [120] clarified that what was reported in the published manuscript was actually SE; COPD = Chronic obstructive pulmonary disease; *SD calculated from reported SE; * post-test data obtained directly from corresponding author; ***reported as steps/week in original article, divided by 7 days here.

#### Controlled studies

Controlled studies conducted on treadmills or designated walking courses can provide direct information about the number of steps in continuous timed walks. The only study identified that focused on older adults was conducted by Tudor-Locke et al. [37] who reported that community-dwelling older adults (mean age 69 years) who were regular exercisers (confirmed by regular attendance at exercise classes that they were recruited from) took approximately 3,400 steps in a 30-minute timed group exercise walk (translating to a cadence or stepping rate of approximately 113 steps/minute) around a gymnasium. Intensity was not directly measured and it is plausible that the group nature of the walk influenced individual paces. However, the finding does fit within estimates for the number of steps taken in 30 minutes of moderate intensity walking in adults [38,39] and within published normal cadence ranges representing "free-speed walking" for men (81-125 steps/minute) and women (96-136 steps/minute) aged 65-80 years [40]. Studies conducted with younger adult samples [41-45] that have directly measured the number of steps and verified activity intensity in absolute terms of metabolic equivalents or METs (1 MET = 3.5 ml O_2_/kg/min or 1 kcal/kg/hour) have concluded that, despite individual variation, a cadence of 100 steps/minute represents a reasonable heuristic value for moderate intensity walking. This suggests that 1,000 steps taken in 10 minutes of walking, or 3,000 steps taken in 30 minutes, could be used to indicate a floor value for absolutely-defined moderate intensity walking. However, it is important to note that this cadence may be unattainable for some individuals living with disability or chronic disease (including frail older adults), reflecting known differences between absolute and relative intensity with age and illness [46]. Unfortunately, there are no data to specifically inform absolute or relative intensity of different cadences in healthy older adults. With that being said, it is possible that *any *increase in daily step count relative to individualized baseline values could confer health benefits. This is congruent with the now accepted concept that some activity is better than none, and that some relatively important health benefits may be realized even with improvements over the lowest levels [5].

In a clinically-based study, 64 older subjects with peripheral artery disease (PAD) and claudication took 575 ± 105 steps to ambulate 355 ± 74 meters during a 6-minute walk test, equating to an average speed of 2.2 mph and an average cadence of 96 steps/min [47]. Given that these research participants were instructed to cover as much distance as possible, this average cadence represents a relatively high exercise intensity (i.e., possibly exceeding moderate intensity, at least in terms of relative intensity) in this population. This is confirmed by the results of a separate study that demonstrated that for these patients, walking at a slightly slower speed of 2.0 mph equates to an energy expenditure of approximately 70% of their peak oxygen uptake [48].

Walking at a cadence of 96 steps/min during a clinical test represents a much higher ambulatory challenge than that measured during free-living daily activities of PAD patients monitored for one week with a step activity monitor [49]. The maximum cadence for one minute of free-living ambulation (i.e., the minute with the single highest cadence value each day) averaged 90.8 steps/min, which was significantly lower than the average value of 99 steps/min in age-matched control subjects from the same study. The maximum cadence for 30 continuous minutes of ambulation each day was only 28 steps/min in PAD patients versus 35.4 steps/min in the age-matched control subjects. Thus, the cadence observed under testing conditions may not be representative of that performed during everyday life.

No other controlled study of cadence or steps taken in timed walks related to intensity was identified for any other special population group. However, the data in older adults with PAD indicate that the relative intensity of walking speeds (captured as cadence) is higher for some groups of older adults, particularly special populations living with disability or chronic illness, than for younger and healthy adults [50,51]. Therefore, future research is needed to extend values for measured cadences, associated walking speeds, absolute intensity (MET values), and ratings of perceived exertion and/or heart rate (to assess relative intensity) in healthy older adults across a range of abilities, as well as in disease-specific populations. Although there appears to be general agreement with regards to the cadence (i.e., 100 steps/min) associated with an absolute measure of moderate intensity in younger adult samples [41-45], it is likely that cadence associated with relative intensity will differ between individuals in much the same manner as heart rate.

### Computed step count translations for physical activity guidelines

Physical activity guidelines from around the world do not generally recommend that older adults do less aerobic activity than younger adults [5,52]. If anything, there seems to be even more emphasis on the importance of obtaining adequate amounts of MVPA over and above activities of daily living [3]. It therefore makes sense to recommend a similar step-based translation of physical activity guidelines for healthy older adults as for their younger counterparts. However, in special populations, specifically individuals (young or old) living with disability and chronic illness, it is important to promote a physically active lifestyle to the fullest extent that it is possible, even if this may fall short of general public health recommendations. For these groups where an absolute intensity or cadence interpretation may not be realistic, a shift to promoting relative intensity (and therefore relative cadence) may become increasingly important to maintain physical function and independence. In essence, for those living at the lowest levels of habitual physical activity, the clinical perspective becomes paramount and overtakes the need for more generic public health messaging.

As noted above, there is no evidence to inform a moderate intensity cadence specific to older adults at this time. However, using the adult cadence of 100 steps/minute to denote the floor of absolutely-defined moderate intensity walking, and multiplying this by 30 minutes, produces an estimate of 3,000 steps. To be a true translation of public health guidelines these steps should be taken over and above activities of daily living, be of at least moderate intensity accumulated in minimally 10 minute bouts, and add up to at least 150 minutes spread out over the week [3,5,53]. Considering a background of daily activity of 5,000 steps/day [15,16], a computed translation of this recommendation produces an estimate of approximately 8,000 on days that include a target of achieving 30 minutes of MVPA, but approximately 7,100 steps/day if averaged over a week (i.e., 7 days at 5,000 plus 15,000 steps of at least moderate intensity). In reality, this background level of daily activity is likely to vary, and it is possible that steps/day values indicative of functional activities of daily living in some older adults (especially special populations living with disability or chronic illness) are much lower than 5,000 steps/day. Recognizing this potential, and as described above, the adult graduated step index has been extended to include 'basal activity' (< 2,500 steps/day) and 'limited activity' (2,500-4,999 steps/day) [17]. Therefore, if we consider 2,500 steps/day as a general indicator of basal activity in older adults and/or individuals living with disability or chronic illness, the minimal estimate is 5,500 daily steps or 4,600 steps/day if averaged over a week of free-living behaviour. Admittedly, these estimates are based on assumed baseline levels, but also an increment that is tied to a cadence that has only been established as an indicator of absolutely-defined moderate-intensity walking in younger adults.

The results of the first computational strategy produce a range of 7,100- 8,000 steps/day that should be compatible with all but the most sedentary older adults (normative data indicate 2,000- 9,000 steps/day) [13,14] and includes criterion referenced values for healthy body mass index (BMI) status related to older women (reviewed below; 8,000 steps/day for 60-94 year old women [54]). However, the limited interventions to date assembled in Table 2 suggest that it may be precisely these most sedentary older adults who are recruited for such pedometer-based interventions. The second strategy produces a range of approximately 4,600- 5,500 steps/day, which seems reasonable for the most sedentary older adults (i.e., those taking < 2,500 steps/day), typically characterized as living with disability and chronic illness, but would under value the achievements of more active older adults or those with chronic illness that does not limit their physical mobility or endurance capacity. Communication using a graduated step index would span these two concerns by providing additional "rungs on the ladder" that take into consideration individual variability while still promoting healthful increases in physical activity. Barring health issues that might compromise abilities, there appears to be no need to otherwise reduce physical activity guidelines for apparently healthy older adults (compared to those for young to middle-aged adults). Any lower accommodation is only in recognition of anyone (including both younger adults *and *older adults) living with disabilities or chronic illness that challenge their physical abilities. It is important to emphasize that both of the computational strategies outlined above produce minimal (or threshold) estimates and it is expected that even more physical activity will be beneficial.

### Direct studies of step equivalents of physical activity guidelines

Rowe et al. [55] studied older adults' (60+ years of age) pedometer-determined steps/day and used a Receiver Operating Curve (ROC) analysis to inform maximal classification accuracy related to 30+ minutes of accelerometer-determined MVPA. They reported that 6,200-6,800 steps/day taken in the course of everyday life was congruent with the time-and intensity-based guidelines if discontinuous (i.e., interrupted) minutes of MVPA were accepted and 7,000-8,000 steps/day if 30 minutes of continuous (bouts ≥ 10 minutes) MVPA was required.

Aoyagi and Shephard [56] reviewed results of a number of studies based on the Nakanajo Study of Older Adults and shared data related to patterns of physical activity collected using an accelerometer (modified Kenz Lifecorder, Suzuken Co., Ltd., Nagoya, Aichi, Japan) that detected both steps and time in MVPA defined as > 3 METs. They reported a strong (*r*^2 ^= .93) correlation between the two outputs such that those who took < 2,000 steps/day spent almost no time in MVPA. From that point, each 1,000 step increment in daily free-living activity up to 6,000 steps/day was associated with an additional 2.5 minutes of MVPA. From 6,000- 12,000 steps/day each 1,000 step increment added another 5 minutes of MVPA. Corresponding increases in MVPA associated with an additional 1,000 steps from 12,000- 18,000 steps/day and above 18,000 steps/day were 7.5 minutes and 10 minutes, respectively. These findings indicate that 30 minutes of MVPA is associated with 10,000 steps/day in older adults (computing a running total from the details reported above). To be clear, although continuous walking performed under laboratory conditions consistently demonstrates that 1,000 steps taken continuously over 10 minutes meets the criterion for absolutely-defined moderate intensity [41-45], step accumulation patterns under free-living conditions include lighter intensity activities and ultimately suggest that substantially more total steps must be accrued in order to achieve recommended amounts of MVPA performed in the course of daily living.

Ayabe et al. [57] also used a Suzuken Lifecorder accelerometer to record both step and physical activity energy expenditure (PAEE) among cardiac rehabilitation patients. Steps/day correlated strongly with PAEE (*r *= .92) and with time spent in MVPA (*r *= .85). Achievement of minimal amounts of recommended PAEE (i.e., 1,500 kcal/week) corresponded with a daily total of 6,470 steps/day and optimal amounts (i.e., 2,200 kcal/week) corresponded with 8,496 steps/day.

In summary, the evidence suggests that, in apparently healthy older adults, taking approximately 7,000-10,000 steps/day under free-living conditions is equivalent to accumulating 30 minutes/day of MVPA (as detected by accelerometer). The only direct evidence of a steps/day equivalent of recommended amounts of MVPA that is specific to any special population (in this case, cardiac rehabilitation patients) indicates that minimal and optimal amounts of PAEE are accumulated with approximately 6,500-8,500 steps/day, respectively. The evidence to support a more specific translation of public health guidelines into steps/day for special populations is lacking. In addition, as presented above, the wide variety and types of disabilities observed in special populations may limit individual ability to perform exercise at any rigidly defined absolute moderate intensity, thus requiring a shift toward clinical strategies focused on relative goal attainment and related improvements.

### Steps/day associated with various health outcomes

Eight cross-sectional studies have focused on older adults. Newton et al. [58] found that accumulating over 7,500 steps/day was related to reduced perceptions of fatigue in older women (mean age 63 years) with a diagnosis of primary biliary cirrhosis. This was the only study of steps/day associated with any health outcome identified in any special population.

Yasunaga et al. [59] split total values of steps/day into quartiles and reported concurrently accumulated time in MVPA (from the same instrument; Suzuken Lifecorder) and health-related quality of life (HRQoL) in older adults. They reported that HRQoL was better in the second quartile of steps/day (men: 5,500 steps/day and 13 minutes detected in moderate intensity; women: 4,500 steps/day and 14 minutes moderate intensity) compared to the first quartile but that no additional benefit (smaller and clinically insignificant improvements) was observed with higher quartiles. Although these were cross-sectional data, the authors suggested that an increase of 2,000 steps over baseline might be recommended for enhanced HRQoL in older adults. Park et al. [60] conducted a similar analysis, this time focused on presence vs. absence of metabolic syndrome in older adults. They reported age-range specific results. They observed a lower likelihood of metabolic syndrome in 65 to 74 year olds who took 10,000 steps/day and/or 30 minutes at > 3 METs (also from the same instrument; Suzuken Lifecorder) and in those > 75 years of age who took 8,000 steps/day and/or 20 minutes at > 3 METs.

Shimuzu et al. [61] studied the effects of habitual physical activity determined using a pedometer on an indicator of immune functioning (salivary secretory immunoglobulin A or sIgA) in older Japanese adults (aged 65-86 years). The steps/day data were split into quartiles and the results showed that older adults who took more than approximately 7,000 steps/day also had the highest level of sIgA and this was significantly higher compared to older adults who took < 4,600 steps/day. Mitsui et al. [62] also studied older (mean age 62.8 years) Japanese adults and reported that women taking 7,500-9,999 steps/day had significantly lower BMI and percent body fat than women taking < 5,000 steps/day. Although this study failed to observe any significant difference between those taking > 10,000 steps/day and those taking < 5,000 steps/day, there were only 14/117 women who took > 10,000 steps/day. Thus, this study was likely underpowered to identify small to modest differences in BMI that might exist. In addition, obesity in these older Japanese women was low (the mean BMI for the sample was 22.2 kg/m^2^). The only significant difference in health parameters observed in men in this study across step-defined physical activity was in triglycerol levels; only men who took > 10,000 steps/day showed significantly lower values.

Foley et al. [63] examined the relationship between pedometer-determined steps/day and bone density at the spine and hip in older adults between 50 and 80 years of age. In men and women over age 65, the increasing difference in hip bone density ranged from 3.1% to 9.4% across the increasing steps/day quartiles. The effect on the spine was only observed in women. There was no threshold effect, that is, bone density continued to be higher with higher steps/day. In a second study of older Japanese women (age 61 to 87 years of age), Kitigawa et al. [64] observed a positive association (adjusted for age and weight) between ultrasound-measured calcaneus bone density and steps/day up to a maximum of 12,000 steps/day; thereafter additional steps/day were not associated with any further increase in bone density.

Tudor-Locke et al. [54] reported an age-specific analysis of BMI-criterion referenced and amalgamated data collected from around the world. For women aged 60-94 years of age the best cut point was 8,000 steps/day in terms of discriminating between BMI-defined normal weight and overweight/obesity. In men aged 51-88 years the value was 11,000 steps/day. The authors acknowledged that they had better confidence in the women's data since the men's value was based on a sparse sample size collected over a relatively wider age range. It is important to note that spring-levered pedometers are known to undercount steps related to obesity [65], so these BMI-referenced values can be questioned. However, even accelerometer-determined steps/day differ in a similar pattern across BMI-defined obesity categories [66]. Since pedometers are more likely to be used in clinical and public health applications, it remains important to present these pedometer-determined data as indicators of expected values in these free-living populations (that include obese individuals).

Swartz et al. [67] conducted a simple analysis, reporting blood pressure and fasting glucose results in older adults split by median pedometer-determined steps/day. They reported that active older adults, defined by having steps/day above the median value of 4,227 steps/day, had lower blood pressure and fasting glucose. Since a simple median split suggests only that "more is better," this study cannot be used to inform the dose response relationship, nor can it be used to identify threshold values of steps/day relative to lower blood pressure or fasting glucose in older adults. Schmidt et al. [68] examined cardiometabolic risk, including measures of waist circumference, systolic blood pressure, fasting glucose, triglyceride, and HDL cholesterol, across the graduated step index in a sample that included older adults. They reported that individuals achieving ≥ 5,000 steps/day had a substantially lower prevalence of adverse cardiometabolic health indicators.

In summary, based on these cross-sectional studies, it appears that 4,500-5,500 steps/day is associated with higher HRQoL scores [59] compared to that associated with better measures of immunity (> 7,000 steps/day [61]), metabolic syndrome (8,000-10,000 steps/day [60]), or BMI-defined weight status (8,000-11,000 steps/day [54,62]). Dose-response relationships may also be modified by sex [62,63]. The dose-response relationship with bone density of the hip and, at least in women, spine, appears to be linear and without threshold values [63]. The evidence indicating distinctly different dose-response curves related to step-defined physical activity is consistent with what was presented at a dose-response symposium [69] and may not be limited to older adults [70]. Of course, prospective and intervention studies are needed to confirm any relationship between steps/day and health outcomes. There is a general lack of any evidence relative to special populations at this time.

## Discussion

Monitoring steps taken is only one of many ways to track physical activity and individuals may prefer to count minutes in activity rather than wear any type of step counting device. Step counting by definition is most relevant to ambulatory activity; however, this is not the only activity that can be performed at health-related intensities. Other examples include cycling and swimming. In addition, public health guidelines categorically recognize the importance of other types of non-ambulatory activity, including resistance training [3,5]. Therefore, the estimates contained herein are limited to translations of physical activity guidelines only in terms of ambulatory activity. For those who swim and cycle (e.g., stationary or recumbent cycling), it may be possible to consider adding 'bonus steps' to daily totals to account for these extra non-ambulatory activities [71]. For example, Miller et al. [71] suggest adding 200 steps for every minute of non-ambulatory activities like cycling or swimming. De Greef et al. [72] have instructed participants in pedometer-based interventions to add 150 steps to their daily total for every minute engaged in cycling and/or swimming.

On face value, a step is the fundamental component of walking; it represents the initiation of body weight transfer and a basic expression of human mobility. Cadence, or steps/minute, is a reasonable indicator of speed [73] and is also related to the intensity of ambulation [41-45], and can theoretically capture the "purposefulness" of ambulatory activity. As steps are accumulated more rapidly and continuously, an individual can be said to be walking purposefully, that is, to get somewhere and/or for exercise. Of course, running is represented at the highest cadences, but this is not likely applicable to many older adults or individuals living with disability or chronic illness. As mentioned above, 100 steps/minutes is a cadence that is growing in acceptance as a heuristic value indicative of walking at an absolutely-defined intensity of 3 MET intensity, at least in younger adults [41-45]. This cadence may be unrealistic for many older adults (especially for those who are more frail) or for those living with disability or chronic illness. It may be useful to embrace a "something is better than nothing" approach [5], or even a "better than usual" approach, in terms of setting relative goals for such special populations.

The correlation between age and preferred walking speed in a population study of older adults 60-86 years of age was -.34 (women) and -.41 (men) [74]. Those living with disability or chronic illness may walk at even slower speeds [75]. Overall, aging, disabled, and ill older adults may gradually lose their ability to walk at higher cadences and what remains is the "pottering" (i.e., random, unplanned movements) associated with activities of daily living that all ages appear to engage in to some extent [76]. Slow walking speed in older adults is strongly associated with increased risk of cardiovascular mortality [77]. Since public health guidelines for older adults continue to emphasize the importance of engagement in aerobic activities that are of at least moderate intensity, it follows that any step count translation also reflects this emphasis. Although pedometers have been widely criticized for not being sensitive to detecting slow walking, their ability to "censor" low force accelerations might actually be seen as a feature that permits a concerted focus on only those steps that are more likely to be beneficial to health [78].

Regardless, the interest in detecting even very low force accelerations is evident from research studies focused on physical activity behaviours of older adults [13,79] and especially of individuals living with disability and chronic illness [14] that have been adopting the StepWatch Activity Monitor (SAM, CYMA Corporation, Mountlake Terrace, WA). The SAM is an ankle worn-accelerometer that detects a "stride" or "gait cycle." To be interpreted relative to more traditional waist-mounted instruments (both accelerometers and pedometers), its output needs to be doubled and expressed as steps. However, this instrument is designed to be exceptionally sensitive to slow gaits [80] (and is also more likely to detect "fidgeting" activities [80]) and therefore its output would appear higher than that of more traditional pedometers [17]. For example, a sample of older adults (mean age 83 years) who wore the SAM for 6 consecutive days averaged approximately 10,000 steps/day [81], or 'active' if directly (and inappropriately) interpreted against the graduated step index based on pedometer output [15,16]. The SAM remains an important research tool, however, it is less practical for public health applications. No conversion factor exists at this time to assist in translation of SAM-detected steps to that of pedometers that have been more traditionally used in research and practice.

Another instrument, the ActiGraph accelerometer, is also known to be more sensitive to lower force accelerations ([82-84]) and its output from earlier models needed to be manipulated in order to interpret it against existing pedometer-based scales [15,16]. More recently, the manufacturers of this instrument have offered a 'low extension' option that can be selected, or deselected, depending on sensitivity requirements. Since pedometers are more likely to be adopted by a range of users including researchers, practitioners, and the general public, and since public health guidelines specifically emphasize MVPA (and not lighter intensity activities), the step-based translations presented in this article are intentionally more reflective of what would be expected from the use of good quality pedometers. Although the need to detect less forceful steps, especially in some clinical populations can be justified, it remains a concern that comparisons between datasets collected with different devices are hampered unless acceptable conversion factors to facilitate such interpretation can be determined.

**Figure 1:**
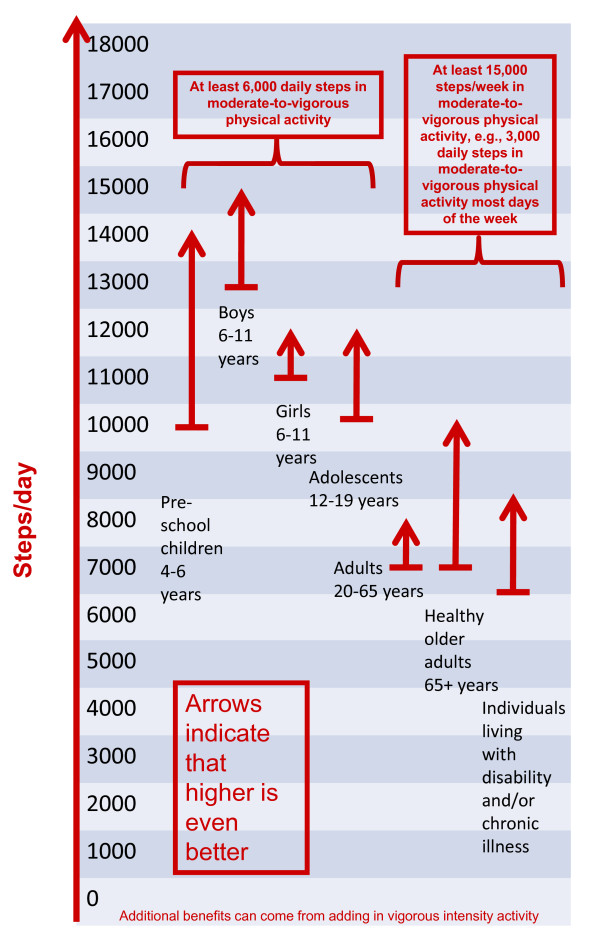
**Steps/day scale schematic linked to time spent in MVPA**.

Regardless of the choice of instrumentation, normative step values for older adults and special populations span a very wide range. Although the graduated step index described above offers a definite improvement over evaluation using any single step value (e.g., 10,000 steps/day), even smaller increments would provide additional "rungs on the ladder" and represent a more continuous and fully expanded steps/day scale. Specifically, 1,000 step increments [41-45] are congruent with the concept of 10-minute bouts taken at 100 steps/min or minimally moderate intensity [3,5], and three 10-minutes bouts (i.e., 3 × 1,000 steps = 3,000 steps) are congruent with a daily 30-minute minimally moderate intensity physical activity recommendation. Figure 1 presents the fully expanded steps/day scale. The scale begins at zero and continues to 18,000+ steps/day, representing the single highest average value reported for a sample at this time in Amish men [85]. Although all age groups are represented, the one-way arrows identify step-based translations of population-specific public health guidelines contained herein (and separately reviewed in companion papers) but also suggest that more is better. For example, the range for healthy older adults is 7,000-10,000 steps/day, at least 3,000 of which should be accumulated at a brisk pace. For individuals living with disability or chronic illness the range is 6,500-8,500 steps/day (although this is based on limited evidence at this time). The difference between thresholds for adults 20-65 years of age and healthy older adults 65+ years of age is nominal (i.e., approximately 300 steps), but it is based on the empirical evidence assembled, and suggests that apparently healthy older adults are capable of achieving minimum steps/day for improving health. However, quite clearly there is a larger gap at the upper end, which reflects decreasing capacity with age (and disease and disability) to achieve upper-end targets. Again, it is important to emphasize, that the oldest-old, especially those compromised by frailty, are more likely to be described as a special population where a clinical approach to increasing physical activity will more appropriately supersede a public health approach. Regardless, adoption of this fully expanded steps/day scale applied across the lifespan would facilitate communication, evaluation, and research. As evidence accumulates, it may be possible to locate population-specific likelihoods of achieving valued health-related outcomes along the scale.

An important limitation must be emphasized. It is well known that the measurement mechanism of accelerometers is more sensitive to lower force accelerations (e.g., slow walking) and therefore this type of instrumentation will detect more steps than simple pedometers. However, there are no data at this time to inform us about the health value of steps taken at very low intensity steps independent of higher intensity steps. Indeed, perhaps one contributory factor to age-related decline is the decrease in intensity of daily movement and the progressive loss of higher intensity movements. This is speculative. Regardless, the difference in instrument sensitivity makes it so that the output of accelerometers should generally not be directly interpreted against the scaling presented herein. A direct conversion factor between instruments is not known at this time, but would certainly be useful. The continued use of BMI as a useful, albeit imperfect, indicator of body fatness is an appropriate analogy to the use of a pedometer as an indicator of healthful levels of physical activity. Regardless, any step-based translation of current physical activity guidelines should clearly convey the importance of making an appropriate portion of daily steps congruent with undertaking recommended amounts and bouts of MVPA [86,87].

## Conclusions

The very broad ranges of habitual activity evident from normative data reflect the natural diversity of physical capacity common to older adults and special populations. There is no evidence to inform an absolutely-defined moderate intensity cadence specific to older adults at this time. However, using the adult cadence of 100 steps/minute to denote the floor of absolutely-defined moderate intensity walking, and multiplying this by 30 minutes produces a reasonable heuristic value of 3,000 steps. To be a true translation of public health guidelines these steps should be taken over and above activities of everyday living, be of at least moderate intensity accumulated in minimally 10 minute bouts (i.e., at least 1,000 steps taken at a cadence of 100 steps/min), and add up to at least 150 minutes spread out over the week. Computed translations of this recommendation approximate 8,000 daily steps and 7,100 steps/day if averaged over a week. Directly measured estimates of free-living activity that include recommended amounts of MVPA are not too different: 7,000 -10,000 steps/day. Recognizing that the most sedentary older adults and individuals living with disability and chronic illness may be more limited in their everyday activities, but could still benefit from a physically active lifestyle, a similarly computed translation approximates 5,500 daily steps or 4,600 steps/day if averaged over a week of free-living behaviour. Direct evidence (measured objectively by accelerometer) suggests a somewhat higher range (6,500- 8,500 steps/day), however, it is important to remember that this is based on a single study of patients in a cardiac rehabilitation program. Direct evidence is urgently needed for other special populations. Individuals living with more physically limiting conditions may demonstrate lower normative values and thus may benefit from more individualized daily step targets relative to their unique baseline values. Health outcome-referenced values of steps/day appear to differ in older adults depending upon which health-related outcome is desired. All estimates herein express translations of minimal recommendations, and more is likely better.

## Competing interests

The following authors declare they have no competing interests: CT-L, YA, RCB, KAC, IDB, BE, AWG, YH, LDL, SMM, FAR-M, LQR, DAR, MDS, and MAT. CLC is associated with the Canadian Fitness and Lifestyle Research Institute which is funded in part by the Public Health Agency of Canada (PHAC). SNB receives book royalties (< $5,000/year) from Human Kinetics; honoraria for service on the Scientific/Medical Advisory Boards for Alere, Technogym, Santech, and Jenny Craig; and honoraria for lectures and consultations from scientific, educational, and lay groups. During the past 5-year period SNB has received research grants from the National Institutes of Health, Department of Defence, Body Media, and Coca Cola.

## Authors' contributions

CT-L and CLC conceived and designed the project. CT-L acquired the data and prepared analysis for initial interpretation. DAR conducted additional analyses. All authors contributed to subsequent interpretation of data. CT-L prepared a draft of the manuscript. All authors contributed to critically revising the manuscript for important intellectual content. KAC, DAR, MDS, and MAT verified data presented in the tables. All authors gave final approval of the version to be published and take public responsibility for its content.

## References

[B1] Chodzko-Zajko WJ Proctor DN Fiatarone Singh MA Minson CT Nigg CR Salem GJ Skinner JS American College of Sports Medicine position stand. Exercise and physical activity for older adults Med Sci Sports Exerc 2009 41 1510 1530 10.1249/MSS.0b013e3181a0c95c 19516148

[B2] Physical Activity Guidelines Advisory Committee Physical Activity Guidelines Advisory Committee Report, 2008 2008 Washington, D.C.: U.S. Department of Health and Human Services

[B3] Paterson DH Warburton DE Physical activity and functional limitations in older adults: a systematic review related to Canada's Physical Activity Guidelines Int J Behav Nutr Phys Act 2010 7 38 20459782 10.1186/1479-5868-7-38 PMC2882898

[B4] Global strategy on diet, physical activity and health: Physical activity and older adults http://www.who.int/dietphysicalactivity/factsheet_olderadults/en/index.html

[B5] U.S. Department of Health and Human Services 2008 Physical Activity Guidelines for Americans: Be Active, Healthy, and Happy! Washington, D.C 2008

[B6] Brownson RC Boehmer TK Luke DA Declining rates of physical activity in the United States: what are the contributors? Annu Rev Public Health 2005 26 421 443 10.1146/annurev.publhealth.26.021304.144437 15760296

[B7] Tudor-Locke C Ham SA Walking behaviors reported in the American Time Use Survey 2003-2005 J Phys Act Health 2008 5 633 647 18820341 10.1123/jpah.5.5.633

[B8] Troiano RP Berrigan D Dodd KW Masse LC Tilert T McDowell M Physical activity in the United States measured by accelerometer Med Sci Sports Exerc 2008 40 181 188 18091006 10.1249/mss.0b013e31815a51b3

[B9] Cyarto EV Myers AM Tudor-Locke C Pedometer accuracy in nursing home and community-dwelling older adults Med Sci Sports Exerc 2004 36 205 209 10.1249/01.MSS.0000113476.62469.98 14767241

[B10] Tudor-Locke C Craig CL Beets MW Belton S Cardon GM Duncan S Hatano Y Lubans DR Olds TS Raustorp A Rowe DA Spence JC Tanaka S Blair SN How many steps/day are enough? For children and adolescents Int J Behav Nutr Phys Act 10.1186/1479-5868-8-78 21798014 PMC3166269

[B11] Tudor-Locke C Craig CL Brown WJ Clemes SA De Cocker K Giles-Corti B Hatano Y Inoue S Matsudo SM Mutrie N Oppert J-M Rowe DA Schmidt MD Schofield GM Spence JC Teixeira PJ Tully MA Blair SN How many steps/day are enough? For adults Int J Behav Nutr Phys Act 10.1186/1479-5868-8-79 21798015 PMC3197470

[B12] Tudor-Locke C Myers AM Methodological considerations for researchers and practitioners using pedometers to measure physical (ambulatory) activity Res Q Exerc Sport 2001 72 1 12 11253314 10.1080/02701367.2001.10608926

[B13] Tudor-Locke C Hart TL Washington TL Expected values for pedometer-determined physical activity in older populations Int J Behav Nutr Phys Act 2009 10.1186/1479-5868-6-59 19706192 PMC3224895

[B14] Tudor-Locke C Washington TL Hart TL Expected values for steps/day in special populations Prev Med 2009 49 3 11 10.1016/j.ypmed.2009.04.012 19409409

[B15] Tudor-Locke C Bassett DR Jr How many steps/day are enough? Preliminary pedometer indices for public health Sports Med 2004 34 1 8 10.2165/00007256-200434010-00001 14715035

[B16] Tudor-Locke C Hatano Y Pangrazi RP Kang M Revisiting "How many steps are enough?" Med Sci Sports Exerc 2008 40 S537 543 10.1249/MSS.0b013e31817c7133 18562971

[B17] Tudor-Locke C Johnson WD Katzmarzyk PT Accelerometer-determined steps per day in US adults Med Sci Sports Exerc 2009 41 1384 1391 10.1249/MSS.0b013e318199885c 19516163

[B18] Ramirez-Marrero FA Rivera-Brown AM Nazario CM Rodriguez-Orengo JF Smit E Smith BA Self-reported physical activity in Hispanic adults living with HIV: comparison with accelerometer and pedometer J Assoc Nurses AIDS Care 2008 19 283 294 10.1016/j.jana.2008.04.003 18598903

[B19] Bravata DM Smith-Spangler C Sundaram V Gienger AL Lin N Lewis R Stave CD Olkin I Sirard JR Using pedometers to increase physical activity and improve health: a systematic review JAMA 2007 298 2296 2304 10.1001/jama.298.19.2296 18029834

[B20] Richardson CR Newton TL Abraham JJ Sen A Jimbo M Swartz AM A meta-analysis of pedometer-based walking interventions and weight loss Ann Fam Med 2008 6 69 77 10.1370/afm.761 18195317 PMC2203404

[B21] Kang M Marshall SJ Barreira TV Lee JO Effect of pedometer-based physical activity interventions: a meta-analysis Res Q Exerc Sport 2009 80 648 655 19791652 10.1080/02701367.2009.10599604

[B22] De Cocker KA De Bourdeaudhuij IM Brown WJ Cardon GM Effects of "10,000 steps Ghent": a whole-community intervention Am J Prev Med 2007 33 455 463 10.1016/j.amepre.2007.07.037 18022061

[B23] Eakin EG Mummery K Reeves MM Lawler SP Schofield G Marshall AJ Brown WJ Correlates of pedometer use: results from a community-based physical activity intervention trial (10,000 Steps Rockhampton) Int J Behav Nutr Phys Act 2007 4 31 10.1186/1479-5868-4-31 17655770 PMC1950707

[B24] Rosenberg D Kerr J Sallis JF Patrick K Moore DJ King A Feasibility and outcomes of a multilevel place-based walking intervention for seniors: a pilot study Health Place 2009 15 173 179 10.1016/j.healthplace.2008.03.010 18502164 PMC6217940

[B25] Croteau KA Richeson NA A matter of health: Using pedometers to increase the physical activity of older adults Activities, Adaptation, and Aging 2005 30 37 47

[B26] Croteau KA Richeson NA Vines SW Jones DB Effects of a pedometer-based physical activity program on older adults' mobility-related self-efficacy and physical performance Activities, Adaptation, and Aging 2004 28 19 33

[B27] Opdenacker J Boen F Coorevits N Delecluse C Effectiveness of a lifestyle intervention and a structured exercise intervention in older adults Prev Med 2008 46 518 524 10.1016/j.ypmed.2008.02.017 18405960

[B28] Mustian KM Peppone L Darling TV Palesh O Heckler CE Morrow GR A 4-week home-based aerobic and resistance exercise program during radiation therapy: a pilot randomized clinical trial J Support Oncol 2009 7 158 167 19831159 PMC3034389

[B29] Tudor-Locke C A preliminary study to determine instrument responsiveness to change with a walking program: physical activity logs versus pedometers Res Q Exerc Sport 2001 72 288 292 11561394 10.1080/02701367.2001.10608962

[B30] Swenson KK Nissen MJ Henly SJ Physical activity in women receiving chemotherapy for breast cancer: adherence to a walking intervention Oncol Nurs Forum 2010 37 321 330 10.1188/10.ONF.321-330 20439216

[B31] Lemaster JW Mueller MJ Reiber GE Mehr DR Madsen RW Conn VS Effect of weight-bearing activity on foot ulcer incidence in people with diabetic peripheral neuropathy: feet first randomized controlled trial Phys Ther 2008 88 1385 1398 10.2522/ptj.20080019 18801859

[B32] Pinto BM Frierson GM Rabin C Trunzo JJ Marcus BH Home-based physical activity intervention for breast cancer patients J Clin Oncol 2005 23 3577 3587 10.1200/JCO.2005.03.080 15908668

[B33] Pinto BM Rabin C Dunsiger S Home-based exercise among cancer survivors: adherence and its predictors Psychooncology 2009 18 369 376 10.1002/pon.1465 19242921 PMC2958525

[B34] Pinto BM Rabin C Abdow S Papandonatos GD A pilot study on disseminating physical activity promotion among cancer survivors: a brief report Psychooncology 2008 17 517 521 10.1002/pon.1268 17847122

[B35] Matthews CE Wilcox S Hanby CL Der Ananian C Heiney SP Gebretsadik T Shintani A Evaluation of a 12-week home-based walking intervention for breast cancer survivors Support Care Cancer 2007 15 203 211 10.1007/s00520-006-0122-x 17001492

[B36] Nguyen HQ Gill DP Wolpin S Steele BG Benditt JO Pilot study of a cell phone-based exercise persistence intervention post-rehabilitation for COPD Int J Chron Obstruct Pulmon Dis 2009 4 301 313 19750190 10.2147/copd.s6643 PMC2740952

[B37] Tudor-Locke C Jones R Myers AM Paterson DH Ecclestone NA Contribution of structured exercise class participation and informal walking for exercise to daily physical activity in community-dwelling older adults Res Q Exerc Sport 2002 73 350 356 12230344 10.1080/02701367.2002.10609031

[B38] Welk GJ Differding JA Thompson RW Blair SN Dziura J Hart P The utility of the Digi-walker step counter to assess daily physical activity patterns Med Sci Sports Exerc 2000 32 S481 488 10.1097/00005768-200009001-00007 10993418

[B39] Wilde BE Soidman CL Corbin CB A 10,000-step count as a physical activity target for sedentary women Res Q Exerc Sport 2001 72 411 414 11770790 10.1080/02701367.2001.10608977

[B40] Whittle MW Gait Analysis: An Introduction 2007 Edinburgh: Elselvier

[B41] Tudor-Locke C Sisson SB Collova T Lee SM Swan PD Pedometer-determined step count guidelines for classifying walking intensity in a young ostensibly healthy population Can J Appl Physiol 2005 30 666 676 10.1139/h05-147 16485518

[B42] Marshall SJ Levy SS Tudor-Locke CE Kolkhorst FW Wooten KM Ji M Macera CA Ainsworth BE Translating physical activity recommendations into a pedometer-based step goal: 3000 steps in 30 minutes Am J Prev Med 2009 36 410 415 10.1016/j.amepre.2009.01.021 19362695

[B43] Beets MW Agiovlasitis S Fahs CA Ranadive SM Fernhall B Adjusting step count recommendations for anthropometric variations in leg length J Sci Med Sport 2010 13 509 512 10.1016/j.jsams.2009.11.002 20096631

[B44] Rowe DA Welk GJ Heil DP Mahar MT Kemble CD Calabro MA Camenisch K Stride rate recommendations for moderate intensity walking Med Sci Sports Exerc 2011 43 312 318 20543754 10.1249/MSS.0b013e3181e9d99a

[B45] Abel M Hannon J Mullineaux D Beighle A Determination of step rate thresholds corresponding to physical activity classifications in adults J Phys Act Health 2011 8 45 51 21297184 10.1123/jpah.8.1.45

[B46] Shephard RJ Absolute versus relative intensity of physical activity in a dose-response context Med Sci Sports Exerc 2001 33 S400 418 discussion S419-420 10.1097/00005768-200106001-00008 11427764

[B47] Montgomery PS Gardner AW The clinical utility of a six-minute walk test in peripheral arterial occlusive disease patients J Am Geriatr Soc 1998 46 706 711 9625185 10.1111/j.1532-5415.1998.tb03804.x

[B48] Gardner AW Ritti-Dias RM Stoner JA Montgomery PS Scott KJ Blevins SM Walking economy before and after the onset of claudication pain in patients with peripheral arterial disease J Vasc Surg 2010 51 628 633 10.1016/j.jvs.2009.09.053 20206808 PMC2842228

[B49] Gardner AW Montgomery PS Scott KJ Afaq A Blevins SM Patterns of ambulatory activity in subjects with and without intermittent claudication J Vasc Surg 2007 46 1208 1214 10.1016/j.jvs.2007.07.038 17919876 PMC2222553

[B50] Jones LM Waters DL Legge M Walking speed at self-selected exercise pace is lower but energy cost higher in older versus younger women J Phys Act Health 2009 6 327 332 19564661 10.1123/jpah.6.3.327

[B51] Mian OS Thom JM Ardigo LP Narici MV Minetti AE Metabolic cost, mechanical work, and efficiency during walking in young and older men Acta Physiol (Oxf) 2006 186 127 139 10.1111/j.1748-1716.2006.01522.x 16497190

[B52] Public Health Agency of Canada and the Canadian Society for Exercise Physiology Canada's Physical Activity Guide to Healthy Active Living for Older Adults 1999 Ottawa, Ont.: Public Health Agency

[B53] O'Donovan G Blazevich AJ Boreham C Cooper AR Crank H Ekelund U Fox KR Gately P Giles-Corti B Gill JM Hamer M McDermott I Murphy M Mutrie N Reilly JJ Saxton JM Stamatakis E The ABC of Physical Activity for Health: a consensus statement from the British Association of Sport and Exercise Sciences J Sports Sci 2010 28 573 591 10.1080/02640411003671212 20401789

[B54] Tudor-Locke C Bassett DR Jr Rutherford WJ Ainsworth BE Chan CB Croteau K Giles-Corti B Le Masurier G Moreau K Mrozek J Oppert JM Raustorp A Strath SJ Thompson D Whitt-Glover MC Wilde B Wojcik JR BMI-referenced cut points for pedometer-determined steps per day in adults J Phys Act Health 2008 5 Suppl 1 S126 139 18364517 10.1123/jpah.5.s1.s126 PMC2866423

[B55] Rowe DA Kemble CD Robinson TS Mahar MT Daily walking in older adults: day-to-day variability and criterion-referenced validity of total daily step counts J Phys Act Health 2007 4 434 446 18209234

[B56] Aoyagi Y Shephard RJ Steps per day: the road to senior health? Sports Med 2009 39 423 438 10.2165/00007256-200939060-00001 19453204

[B57] Ayabe M Brubaker PH Dobrosielski D Miller HS Kiyonaga A Shindo M Tanaka H Target step count for the secondary prevention of cardiovascular disease Circ J 2008 72 299 303 10.1253/circj.72.299 18219170

[B58] Newton JL Bhala N Burt J Jones DEJ Characterisation of the associations and impact of symptoms in primary biliary cirrhosis using a disease specific quality of life measure J Hepatol 2006 44 776 783 10.1016/j.jhep.2005.12.012 16487619

[B59] Yasunaga A Togo F Watanabe E Park H Shephard RJ Aoyagi Y Yearlong physical activity and health-related quality of life in older Japanese adults: the Nakanojo Study J Phys Act Health 2006 14 288 301 10.1123/japa.14.3.288 17090806

[B60] Park S Park H Togo F Watanabe E Yasunaga A Yoshiuchi K Shephard RJ Aoyagi Y Year-long physical activity and metabolic syndrome in older Japanese adults: cross-sectional data from the Nakanojo Study J Gerontol A Biol Sci Med Sci 2008 63 1119 1123 18948564 10.1093/gerona/63.10.1119

[B61] Shimizu K Kimura F Akimoto T Akama T Kuno S Kono I Effect of free-living daily physical activity on salivary secretory IgA in elderly Med Sci Sports Exerc 2007 39 593 598 10.1249/mss.0b013e318031306d 17414795

[B62] Mitsui T Shimaoka K Tsuzuku S Kajioka T Sakakibara H Pedometer-determined physical activity and indicators of health in Japanese adults J Physiol Anthropol 2008 27 179 184 10.2114/jpa2.27.179 18832781

[B63] Foley S Quinn S Jones G Pedometer determined ambulatory activity and bone mass: a population-based longitudinal study in older adults Osteoporos Int 2009 10.1007/s00198-009-1137-1 19997903

[B64] Kitagawa J Omasu F Nakahara Y Effect of daily walking steps on ultrasound parameters of the calcaneus in elderly Japanese women Osteoporos Int 2003 14 219 224 12730771 10.1007/s00198-002-1339-2

[B65] Crouter SE Schneider PL Bassett DR Jr Spring-levered versus piezo-electric pedometer accuracy in overweight and obese adults Med Sci Sports Exerc 2005 37 1673 1679 10.1249/01.mss.0000181677.36658.a8 16260966

[B66] Tudor-Locke C Brashear MM Johnson WD Katzmarzyk PT Accelerometer profiles of physical activity and inactivity in normal weight, overweight, and obese U.S. men and women Int J Behav Nutr Phys Act 2010 7 60 10.1186/1479-5868-7-60 20682057 PMC2924256

[B67] Swartz AM Strath SJ Parker SJ Miller NE The impact of body-mass index and steps per day on blood pressure and fasting glucose in older adults J Aging Phys Act 2008 16 188 200 18483441 10.1123/japa.16.2.188

[B68] Schmidt MD Cleland VJ Shaw K Dwyer T Venn AJ Cardiometabolic risk in younger and older adults across an index of ambulatory activity Am J Prev Med 2009 37 278 284 10.1016/j.amepre.2009.05.020 19765498

[B69] Bouchard C Physical activity and health: introduction to the dose-response symposium Med Sci Sports Exerc 2001 33 S347 350 10.1097/00005768-200106001-00002 11427758

[B70] Dwyer T Hosmer D Hosmer T Venn AJ Blizzard CL Granger RH Cochrane JA Blair SN Shaw JE Zimmet PZ Dunstan D The inverse relationship between number of steps per day and obesity in a population-based sample: the AusDiab study Int J Obes (Lond) 2007 31 797 804 10.1038/sj.ijo.0803472 17047641

[B71] Miller R Brown W Tudor-Locke C But what about swimming and cycling? How to 'count' non-ambulatory activity when using pedometers to assess physical activity J Phys Act Health 2006 3 257 266 10.1123/jpah.3.3.257 28834503

[B72] De Greef K Deforche B Tudor-Locke C De Bourdeaudhuij I A cognitive-behavioural pedometer-based group intervention on physical activity and sedentary behaviour in individuals with type 2 diabetes Health Educ Res 2010 10.1093/her/cyq017 PMC2936553 20338978

[B73] Paroczai R Kocsis L Analysis of human walking and running parameters as a function of speed Technol Health Care 2006 14 251 260 17065748

[B74] Callisaya ML Blizzard L Schmidt MD McGinley JL Srikanth VK Ageing and gait variability--a population-based study of older people Age Ageing 2010 39 191 197 10.1093/ageing/afp250 20083617

[B75] Gardner AW Forrester L Smith GV Altered gait profile in subjects with peripheral arterial disease Vasc Med 2001 6 31 34 11358158

[B76] Tudor-Locke C Johnson WD Katzmarzyk PT Relationship between accelerometer-determined steps/day and other accelerometer outputs in U.S. adults J Phys Act Health 2011 8 410 419 21487141 10.1123/jpah.8.3.410

[B77] Dumurgier J Elbaz A Ducimetiere P Tavernier B Alperovitch A Tzourio C Slow walking speed and cardiovascular death in well functioning older adults: prospective cohort study BMJ 2009 339 b4460 10.1136/bmj.b4460 19903980 PMC2776130

[B78] Tudor-Locke C Lutes L Why do pedometers work? A reflection upon the factors related to successfully increasing physical activity Sports Med 2009 39 981 993 10.2165/11319600-000000000-00000 19902981

[B79] Tudor-Locke C Hart TL Washington TL Correction: Expected values for pedometer-determined physical activity in older populations Int J Behav Nutr Phys Act 2009 6 65 10.1186/1479-5868-6-65 19818122 PMC2765970

[B80] Karabulut M Crouter SE Bassett DR Jr Comparison of two waist-mounted and two ankle-mounted electronic pedometers Eur J Appl Physiol 2005 95 335 343 10.1007/s00421-005-0018-3 16132120

[B81] Cavanaugh JT Coleman KL Gaines JM Laing L Morey MC Using step activity monitoring to characterize ambulatory activity in community-dwelling older adults J Am Geriatr Soc 2007 55 120 124 10.1111/j.1532-5415.2006.00997.x 17233695

[B82] Le Masurier GC Lee SM Tudor-Locke C Motion sensor accuracy under controlled and free-living conditions Med Sci Sports Exerc 2004 36 905 910 15126728 10.1249/01.mss.0000126777.50188.73

[B83] Le Masurier GC Tudor-Locke C Comparison of pedometer and accelerometer accuracy under controlled conditions Med Sci Sports Exerc 2003 35 867 871 10.1249/01.MSS.0000064996.63632.10 12750599

[B84] Tudor-Locke C Ainsworth BE Thompson RW Matthews CE Comparison of pedometer and accelerometer measures of free-living physical activity Med Sci Sports Exerc 2002 34 2045 2051 10.1097/00005768-200212000-00027 12471314

[B85] Bassett DR Jr Schneider PL Huntington GE Physical activity in an Old Order Amish community Med Sci Sports Exerc 2004 36 79 85 10.1249/01.MSS.0000106184.71258.32 14707772

[B86] Johnson ST Boulé NG Bell GJ Bell RC Walking: a matter of quantity and quality physical activity for type 2 diabetes management Appl Physiol Nutr Metab 2008 33 797 801 10.1139/H08-055 18641725

[B87] Johnson ST McCargar LJ Bell GJ Tudor-Locke C Harber VJ Bell RC Walking faster: distilling a complex prescription for type 2 diabetes management through pedometry Diabetes Care 2006 29 1654 1655 10.2337/dc06-0761 16801594

[B88] Ewald B Duke J Thakkinstian A Attia J Smith W Physical activity of older Australians measured by pedometry Australas J Ageing 2009 28 127 133 10.1111/j.1741-6612.2009.00372.x 19845652

[B89] Conn VS Burks KJ Minor MA Mehr DR Randomized trial of 2 interventions to increase older women's exercise Am J Health Behav 2003 27 380 388 12882432 10.5993/ajhb.27.4.10

[B90] Jensen GL Roy MA Buchanan AE Berg MB Weight loss intervention for obese older women: improvements in performance and function Obes Res 2004 12 1814 1820 10.1038/oby.2004.225 15601977

[B91] Croteau KA Richeson NE Farmer BC Jones DB Effect of a pedometer-based intervention on daily step counts of community-dwelling older adults Res Q Exerc Sport 2007 78 401 406 18274211 10.1080/02701367.2007.10599439

[B92] Sarkisian CA Prohaska TR Davis C Weiner B Pilot test of an attribution retraining intervention to raise walking levels in sedentary older adults J Am Geriatr Soc 2007 55 1842 1846 10.1111/j.1532-5415.2007.01427.x 17979902

[B93] Wellman NS Kamp B Kirk-Sanchez NJ Johnson PM Eat Better & Move More: a community-based program designed to improve diets and increase physical activity among older Americans Am J Public Health 2007 97 710 717 10.2105/AJPH.2006.090522 17329647 PMC1829349

[B94] Culos-Reed SN Stephenson L Doyle-Baker PK Dickinson JA Mall walking as a physical activity option: results of a pilot project Can J Aging 2008 27 81 87 10.3138/cja.27.1.81 18492639

[B95] Fitzpatrick SE Reddy S Lommel TS Fischer JG Speer EM Stephens H Park S Johnson MA Physical activity and physical function improved following a community-based intervention in older adults in Georgia senior centers J Nutr Elder 2008 27 135 154 10.1080/01639360802060223 18928194

[B96] Sugden JA Sniehotta FF Donnan PT Boyle P Johnston DW McMurdo ME The feasibility of using pedometers and brief advice to increase activity in sedentary older women--a pilot study BMC Health Serv Res 2008 8 169 18691392 10.1186/1472-6963-8-169 PMC2527003

[B97] Koizumi D Rogers NL Rogers ME Islam MM Kusunoki M Takeshima N Efficacy of an accelerometer-guided physical activity intervention in community-dwelling older women J Phys Act Health 2009 6 467 474 19842461 10.1123/jpah.6.4.467

[B98] Wilson DB Porter JS Parker G Kilpatrick J Anthropometric changes using a walking intervention in African American breast cancer survivors: a pilot study Prev Chronic Dis 2005 2 A16 15888227 PMC1327710

[B99] Vallance JK Courneya KS Plotnikoff RC Yasui Y Mackey JR Randomized controlled trial of the effects of print materials and step pedometers on physical activity and quality of life in breast cancer survivors J Clin Oncol 2007 25 2352 2359 10.1200/JCO.2006.07.9988 17557948

[B100] Irwin ML Cadmus L Alvarez-Reeves M O'Neil M Mierzejewski E Latka R Yu H Dipietro L Jones B Knobf MT Chung GG Mayne ST Recruiting and retaining breast cancer survivors into a randomized controlled exercise trial: the Yale Exercise and Survivorship Study Cancer 2008 112 2593 2606 10.1002/cncr.23446 18428192 PMC5450159

[B101] Blaauwbroek R Bouma MJ Tuinier W Groenier KH de Greef MH Meyboom-de Jong B Kamps WA Postma A The effect of exercise counselling with feedback from a pedometer on fatigue in adult survivors of childhood cancer: a pilot study Support Care Cancer 2009 17 1041 1048 10.1007/s00520-008-0533-y PMC2707951 19015892

[B102] de Blok BM de Greef MH ten Hacken NH Sprenger SR Postema K Wempe JB The effects of a lifestyle physical activity counseling program with feedback of a pedometer during pulmonary rehabilitation in patients with COPD: a pilot study Patient Educ Couns 2006 61 48 55 10.1016/j.pec.2005.02.005 16455222

[B103] Hospes G Bossenbroek L Ten Hacken NH van Hengel P de Greef MH Enhancement of daily physical activity increases physical fitness of outclinic COPD patients: results of an exercise counseling program Patient Educ Couns 2009 75 274 278 10.1016/j.pec.2008.10.005 19036552

[B104] VanWormer JJ Boucher JL Pronk NP Thoennes JJ Lifestyle behavior change and coronary artery disease: effectiveness of a telephone-based counseling program J Nutr Educ Behav 2004 36 333 334 10.1016/S1499-4046(06)60406-5 15617619

[B105] Izawa KP Watanabe S Omiya K Hirano Y Oka K Osada N Iijima S Effect of the self-monitoring approach on exercise maintenance during cardiac rehabilitation: a randomized, controlled trial Am J Phys Med Rehabil 2005 84 313 321 10.1097/01.PHM.0000156901.95289.09 15829777

[B106] Tudor-Locke C Bell RC Myers AM Harris SB Ecclestone NA Lauzon N Rodger NW Controlled outcome evaluation of the First Step Program: a daily physical activity intervention for individuals with type II diabetes Int J Obes Relat Metab Disord 2004 28 113 119 10.1038/sj.ijo.0802485 14569279

[B107] Araiza P Hewes H Gashetewa C Vella CA Burge MR Efficacy of a pedometer-based physical activity program on parameters of diabetes control in type 2 diabetes mellitus Metabolism 2006 55 1382 1387 10.1016/j.metabol.2006.06.009 16979410

[B108] Engel L Lindner H Impact of using a pedometer on time spent walking in older adults with type 2 diabetes Diabetes Educ 2006 32 98 107 10.1177/0145721705284373 16439498

[B109] Richardson CR Mehari KS McIntyre LG Janney AW Fortlage LA Sen A Strecher VJ Piette JD A randomized trial comparing structured and lifestyle goals in an internet-mediated walking program for people with type 2 diabetes Int J Behav Nutr Phys Act 2007 4 59 10.1186/1479-5868-4-59 18021411 PMC2212636

[B110] Bjorgaas MR Vik JT Stolen T Lydersen S Grill V Regular use of pedometer does not enhance beneficial outcomes in a physical activity intervention study in type 2 diabetes mellitus Metabolism 2008 57 605 611 10.1016/j.metabol.2007.12.002 18442621

[B111] Cheong SH McCargar LJ Paty BW Tudor-Locke C Bell RC The First Step First Bite Program: guidance to increase physical activity and daily intake of low-glycemic index foods J Am Diet Assoc 2009 109 1411 1416 10.1016/j.jada.2009.05.012 19631048

[B112] Johnson ST Bell GJ McCargar LJ Welsh RS Bell RC Improved cardiovascular health following a progressive walking and dietary intervention for type 2 diabetes Diabetes Obes Metab 2009 11 836 843 10.1111/j.1463-1326.2009.01050.x 19614943

[B113] Kirk A Barnett J Leese G Mutrie N A randomized trial investigating the 12-month changes in physical activity and health outcomes following a physical activity consultation delivered by a person or in written form in Type 2 diabetes: Time2Act Diabet Med 2009 26 293 301 10.1111/j.1464-5491.2009.02675.x 19317825

[B114] Newton KH Wiltshire EJ Elley CR Pedometers and text messaging to increase physical activity: randomized controlled trial of adolescents with type 1 diabetes Diabetes Care 2009 32 813 815 10.2337/dc08-1974 19228863 PMC2671105

[B115] Tudor-Locke C Lauzon N Myers AM Bell RC Chan CB McCargar LJ Speechley M Rodger NW Effectiveness of the First Step Program delivered by professionals versus peers J Phys Act Health 2009 6 456 462 19842459 10.1123/jpah.6.4.456

[B116] Vincent D Culturally tailored education to promote lifestyle change in Mexican Americans with type 2 diabetes J Am Acad Nurse Pract 2009 21 520 527 10.1111/j.1745-7599.2009.00439.x 19845810

[B117] Diedrich A Munroe DJ Romano M Promoting physical activity for persons with diabetes Diabetes Educ 2010 36 132 10.1177/0145721709352382 20019197

[B118] Talbot LA Gaines JM Huynh TN Metter EJ A home-based pedometer-driven walking program to increase physical activity in older adults with osteoarthritis of the knee: a preliminary study J Am Geriatr Soc 2003 51 387 392 10.1046/j.1532-5415.2003.51113.x 12588583

[B119] Kilmer DD Wright NC Aitkens S Impact of a home-based activity and dietary intervention in people with slowly progressive neuromuscular diseases Arch Phys Med Rehabil 2005 86 2150 2156 10.1016/j.apmr.2005.07.288 16271563

[B120] Fontaine KR Haaz S Effects of lifestyle physical activity on health status, pain, and function in adults with fibromyalgia syndrome J Musculoskelet Pain 2007 15 3 13

